# Solidly mounted resonator sensor for biomolecule detections

**DOI:** 10.1039/c9ra01695c

**Published:** 2019-07-09

**Authors:** Chengzhang Han, Xia Wang, Qiuling Zhao, Lihua Teng, Shuaiyi Zhang, Hao Lv, Jing Liu, Haoran Ma, Yanping Wang

**Affiliations:** Optoelectronic Materials and Technologies Engineering Laboratory, Shandong, Physics Department, Qingdao University of Science and Technology Qingdao 266042 China phwangxia@163.com sdqlzhao@163.com; College of Mechanical and Electronic Engineering, Qingdao Binhai University Qingdao 266555 China

## Abstract

We report the fabrication of a solidly mounted resonator (SMR) that can also function as a sensor for biological molecules. The SMR, consisting of a Au electrode, aluminum nitride (AlN) piezoelectric thin film and Bragg acoustic reflector, was fabricated on a Si substrate by radio frequency (RF) magnetron sputtering. The Bragg acoustic reflector, made entirely of metal, has small internal stress and good heat conduction. Human immunoglobulin G (IgG) antibody was immobilized on the modified (by self-assembled monolayer method) Au electrode surface of the SMR and goat anti-human IgG antigen was captured through the specificity of bond between the antibody and antigen on the electrode surface. We found a linear relationship between the resonant frequency shift and the concentration of goat anti-human IgG antigen for concentrations smaller than 0.4 mg ml^−1^ and a relatively constant frequency shift for concentrations greater than 0.5 mg ml^−1^. A series of interference experiments can prove that the selectivity of the sensor is satisfactory. Our findings suggest that the SMR sensor is an attractive alternative for biomolecule detection.

## Abstract 2

We report the fabrication of a solidly mounted resonator (SMR) that can also function as a sensor for biological molecules.

## Graphical abstract

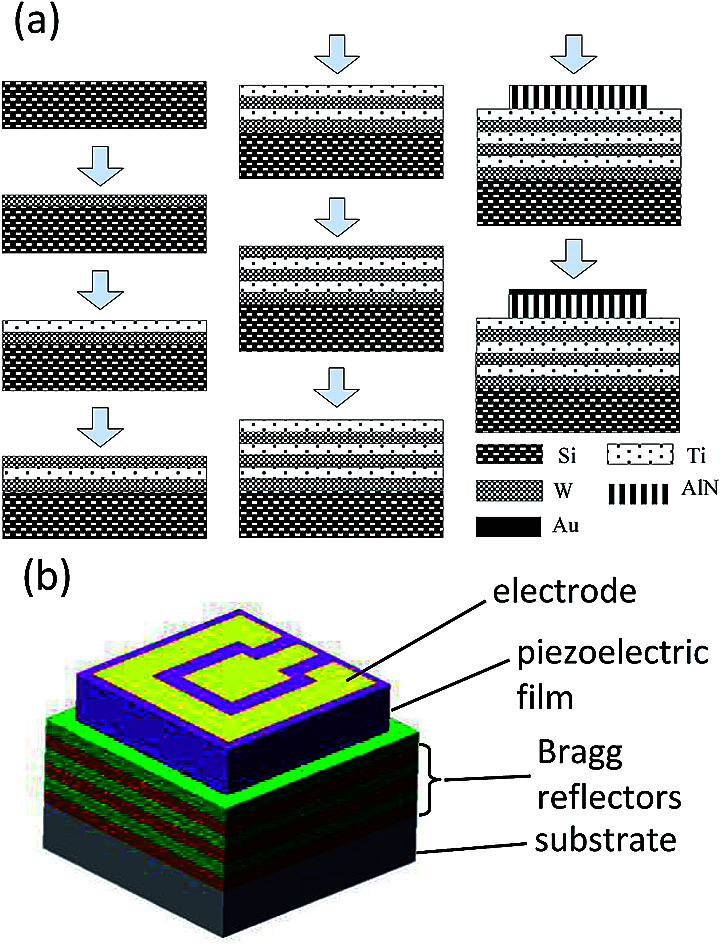

## 1. Introduction

With the advances in molecular biochemical studies, analysis of biomolecular interactions such as antigen–antibody binding, protein–ligand interactions and enzyme–substrate inter-actions plays a vital role in medical diagnostics and environmental protection.^1–4^ Therefore, there are considerable demands in developing small size, high sensitivity, low sample amount and on-chip integration sensors for biomolecular detection.^5,6^ In recent years, various types of sensors such as optical,^7^ electrochemical^8^ and electromechanical^9^ devices have been proposed as biochemical sensors. Among the various detection technologies, mass-sensitive sensors based on piezoelectric resonators have attracted considerable attention for biomolecule detection during the last few decades due to their high selectivity and sensitivity.^10,11^

In generally, these piezoelectric resonator sensors can be divided into two categories (determined by the transmission paths of acoustic wave resonance) surface acoustic wave (SAW) and bulk acoustic wave (BAW) sensors. However, the SAW sensors cannot achieve a higher sensitivity due to the restriction on dimension of the delay line for the interdigital transducer (IDT) and the resonant frequency (from 30 MHz to 1 GHz).^12,13^ In addition, the quartz crystal microbalance (QCM) employed in BAW sensors has a mass detection limit in the order of nanograms determined from their low operation frequency (5–20 MHz) due to the bulk quartz substrate thickness. Thus BAW sensors do not have adequate sensitivity for the detection of small molecules in low concentrations.^14,15^ With the advancement in micro/nano fabrications, film bulk acoustic resonator (FBAR) is proposed as a typical MEMS piezoelectrical device. Importantly FBAR operating in the GHz range can overcome the shortcomings of BAW sensors and have better performance in mass-sensitive detections.^16^ In addition to the sensitivity, FBAR has several other advantages, including small in size, room-temperature operation, and mass producible.^17–19^

Piezoelectric materials such as zinc oxide (ZnO)^20^ and aluminum nitride (AlN)^21^ have been used in FBAR devices for various applications owing to their high acoustic velocity, better quality factor, and high electromechanical coupling coefficient.^22–24^ There are two types of FBARs, one with a freestanding membrane and the other solidly mounted resonator (SMR) composed of a piezoelectric thin film sandwiched between electrodes and Bragg reflector consisting of alternating high and low acoustic impedance quarter-wavelength thick dielectric or metallic layers.^25,26^ By comparing and studying, the SMR with the simple fabrication, good mechanical strength, excellent acoustic properties and closer to CMOS integration was fabricated as a promising candidate for biomolecule detections.^27,28^

In this paper, we explored the possibility of AlN film SMR as a sensor for biomolecule detections. We have fabricated SMR device, immobilized human immunoglobulin G (IgG) antibody on the SMR electrode surface modified by self-assembled monolayer (SAM) method for the detections of goat anti-human IgG antigen.^29^ The sensitivity and usability of sensor were evaluated for goat anti-human IgG antigen detection. In addition, the relationship between the resonant frequency shift of SMR sensor and the concentration of goat anti-human IgG antigen was also investigated.

## 2. Experiment

### 2.1 Reagents and materials

All chemicals and solvents were of reagent grade or better. 11-Mercaptoundecanoic acid (11-MUA), 1-(3-dimethylaminopropyl)-3-ethylcarbodiimide hydrochloride (EDC), *N*-hydroxysuccinimide (NHS) and bovine serum albumin (BSA) were purchased from Sigma-Aldrich. Human IgG, goat anti-human IgG, goat anti-mouse IgG and goat anti-rabbit IgG were purchased from Shanghai Dingguo Biotechnology Co., Ltd (Shanghai, China). The supporting electrolyte was 0.1 M phosphate buffer solution (PBS) prepared with Na_2_HPO_4_ and KH_2_PO_4_. Doubly distilled water was used for preparing all the solutions.

### 2.2 Apparatus

The crystalline structure of the SMR was investigated by X-ray diffraction (XRD, Bruker Advanced D8) using a Cu-Kα radiation (*λ* = 1.54187 Å) in a *θ*–2*θ* scanning mode. The cross-sectional morphology of SMR was observed using a field emission-scanning electron microscope (FE-SEM, Carl Zeiss Ultra55). The sputtering was carried out by a multifunctional nano preparation system (Yuhua CS-350). Finally, the frequency response of SMR was measured by *S*-scattering parameters with a probe station (Cascade EPS 150 RF) and a network analyzer (HP 8712E). All measurements of response of the SMR were carried out in an ambient temperature kept at 25 °C.

### 2.3 SMR fabrication

The procedures of the SMR fabrication were be divided into three steps: Bragg reflector deposition, AlN thin film fabrication, and top Au electrode deposition. Before the formal fabrication, the process parameters of AlN thin film were optimized repeatedly to obtain high *c*-axis orientational quality.^30^ The Bragg reflector, consisting of titanium (Ti) and tungsten (W) layers, was first deposited on p-type 3 inch Si (100) substrate with 1–10 Ω cm resistivity at 25 °C, and then a AlN thin film was deposited on the top of the Bragg reflector at 300 °C. Lastly, a Au film was deposited on top of the AlN for the electrodes. The AlN thin film was obtained by a RF reactive magnetron sputtering system with an Al target in a N_2_ and Ar mixture atmosphere. Similar to the AlN, Ti, W, and Au film were also obtained by the RF magnetron sputtering system using Ti, W, and Au targets in a pure Ar atmosphere, respectively, with about a 70 mm target-to-substrate distance. The purities of all the targets are 99.999% and the diameters of all targets are 80 mm. Finally, a thermal annealing process at 300 °C was also performed to relieve the stress in multilayer films to improve the performance of the SMR.

**Fig. 1:**
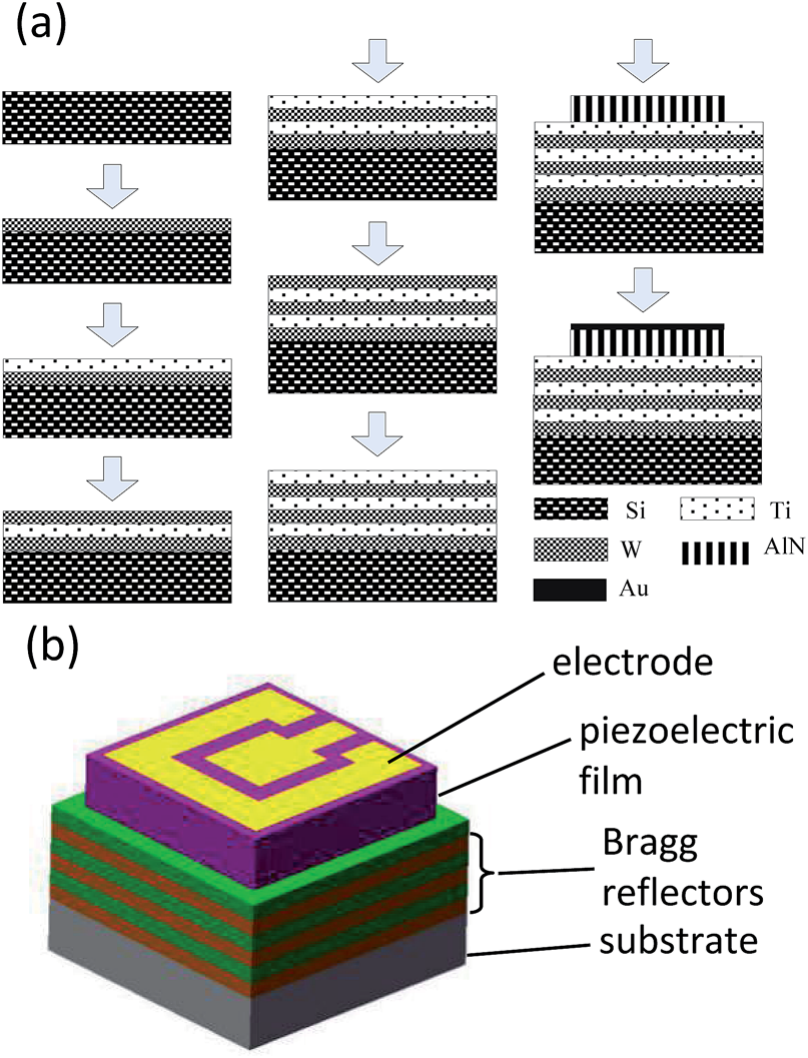
The fabrication process flow and schematic illustration of SMR.

The specific sputtering parameters for different materials in our experiment are summarized in Table 1. The fabrication process flow and schematic illustration of SMR are shown in Fig. 1.

**Table 1:** The specific sputtering parameters of Ti, W, AlN and Au

Sputtering parameters	Ti	W	AlN	Au
Target	Ti (99.999%)	W (99.999%)	Al (99.999%)	Au (99.999%)
RF power (W)	150	150	250	150
Ar flow rate (sccm)	20	20	20	20
N_2_ flow rate (sccm)	0	0	20	0
Sputtering pressure (Pa)	1.5	1.5	1.5	1.5
Substrate temperature (°C)	25	25	300	25
Deposition thickness (nm)	630	570	2360	120

### 2.4 Biomolecules immobilization

The biomolecule immobilization is a very vital step for a SMR sensor as it has dramatic effects on the selectivity and sensibility of sensor. The most popular biomolecule immobilization method is SAM as the cross-linker between biomolecules and the Au electrode surface. The SAM technique offers a simple way to form a fast, reproducible, ultrathin and well-ordered layer suitable for further modification.^31,32^ To start, the electrode surface was pretreated sequentially with piranha solution (H_2_SO_4_ : H_2_O_2_ = 3 : 1) for 5 min, acetone for 5 min and ethanol for 5 min to obtain a clean and hydrophilic gold surface. Then the immobilized surface was washed repeatedly with distilled water and dried with pure N_2_. After that, the resonance frequency *f*_0_ of the SAM was recorded as a reference. To continue the experiment, the electrode surface was covered with a monolayer of 11-MUA by immersed in 1 mmol l^−1^ 11-MUA ethanolic solution for 24 h at room temperature. Then, the electrode surface was washed repeatedly with ethanol and distilled water in sequence and dried with pure N_2_. After that, 0.2 mol l^−1^ EDC and 0.05 mol l^−1^ NHS solutions were mixed uniformity and applied onto the electrode surface for 1 h at room temperature to further activate the electrode surface. Then, 1 μl of 0.5 mg ml^−1^ human IgG antibody was applied onto the surface of SMR for 2 h at 37 °C to achieve complete binding between the human IgG antibody and the modified electrode surface. Again, the electrode surface was washed repeatedly with PBS and distilled water in sequence to remove physically adsorbed human IgG antibody and dried with pure N_2_. To complete the process, the electrode surface was immersed in 10 mg ml^−1^ BSA solution for 2 h at 37 °C subsequently to apply onto antibody-coated to block the unreacted sites and minimize unspecific effects.^33^ After that, a frequency measurement of SMR was carried out again and recorded as *f*_1_.

**Fig. 2:**
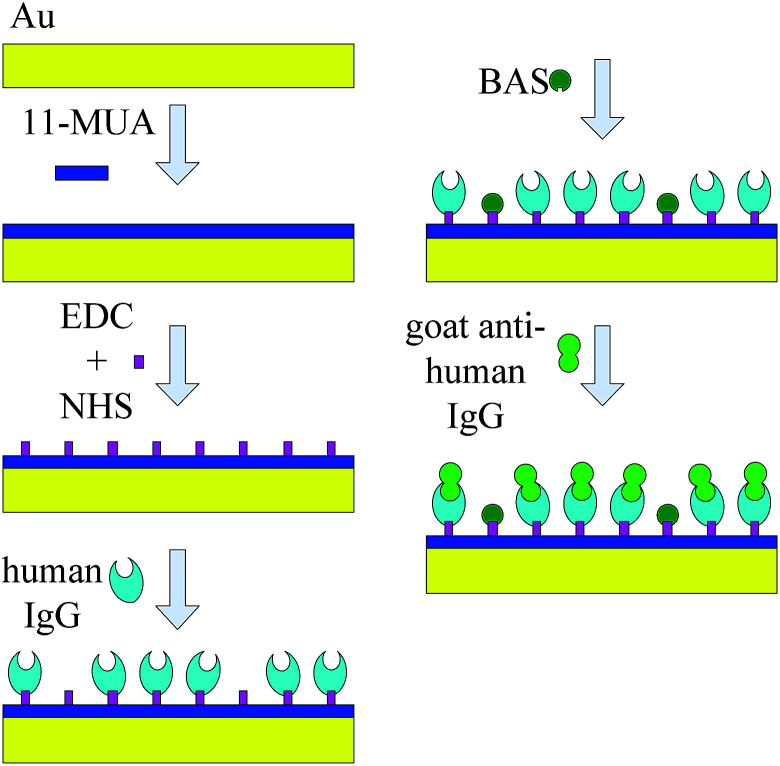
The schematic illustration of biomolecules immobilization on electrode surface of SMR sensor.

For detecting the sensibility and selectivity of the SMR sensor, an amount of 1 μl of 0.5 mg ml^−1^ goat anti-human IgG antigen with fluorescein isothiocyanate (FITC) was applied onto the electrode surface coated with human IgG antibody for 2 h at 37 °C in the dark to achieve complete specific binding between the goat anti-human IgG antigen and human IgG antibody. Then, the electrode surface was washed repeatedly with PBS and distilled water in sequence to remove physically adsorbed goat anti-human IgG antigen and dried with pure N_2_. Finally, a frequency characteristic of SMR was measured again and recorded as *f*_2_. The goat anti-human IgG antigen with FITC immobilization on the electrode surface was confirmed by fluorescence interference microscope (FIM) imaging. The schematic illustration of biomolecules immobilization on the electrode surface of SMR is shown in Fig. 2.

## 3. Results and discussions

**Fig. 3:**
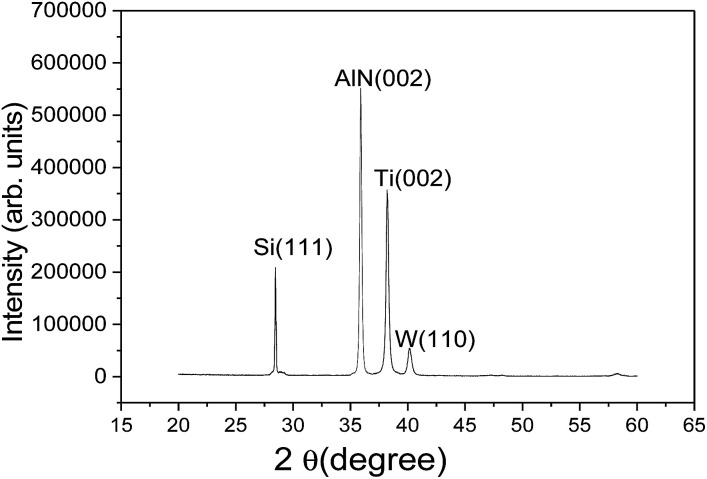
The XRD patterns of SMR grown on silicon substrate.

Fig. 3 shows the XRD patterns of the SMR grown on silicon substrate. The diffraction peaks of silicon, Ti, W, and AlN can be observed clearly from the figure, confirming the structural integrity of SMR fabricated. Specially, for the AlN thin film, a very strong diffraction peak was observed at 35.94° with a full width at half-maximum (FWHM) of 0.30°, which corresponds to the diffraction from the AlN (002) plane. This verified that the preferential AlN growth orientation was along the wurtzite *c*-axis and perpendicular to the surface of substrate. In addition, according to the well-known Scherrer formula,^34^ the crystalline grain size of AlN was calculated to be about 28 nm.

**Fig. 4:**
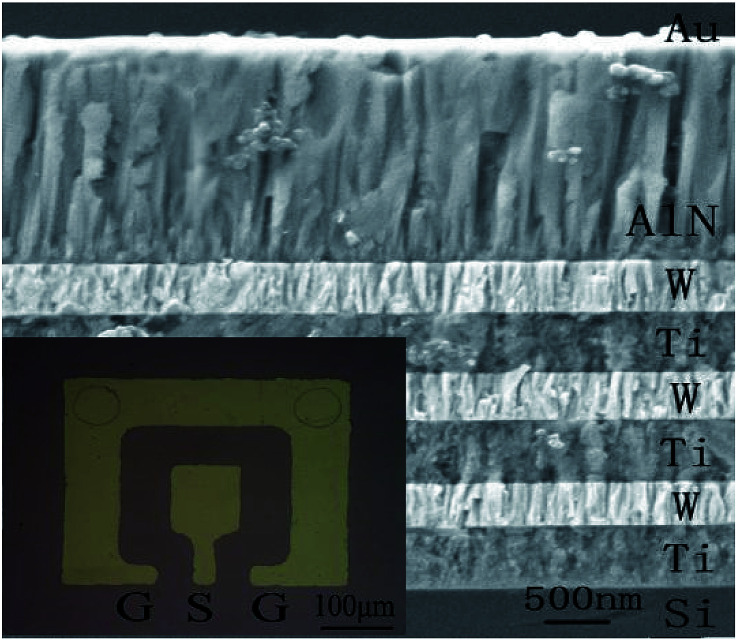
The cross-section view morphologies of the integrated SMR. The inset is a top electrode pattern.

Fig. 4 shows the cross-section view morphologies of the integrated SMR with Au/AlN/Ti/W layered structure and the inset is the top electrode pattern. The AlN film perpendicular to the Bragg reflector exhibits highly oriented and compact columnar structure. The interfaces between the AlN film and Bragg reflector are clearly visible and distinct, verifying that the different membrane layers are not diffusive with each other. As the Bragg reflector was made entirely of metal, it had small internal stress and good heat conduction. Note that bottom Ti layer of the Bragg reflector on the top of Si also served as the electrode for frequency measurements.

**Fig. 5:**
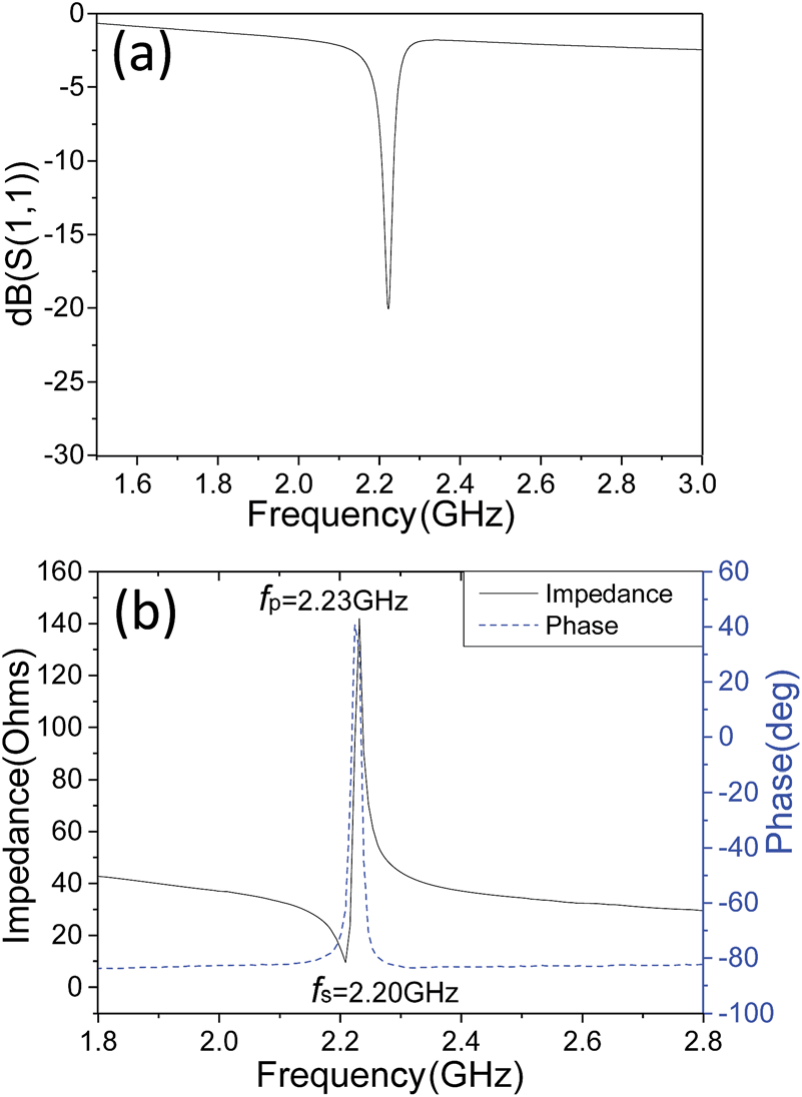
The reflection coefficient *S*(1,1), impedance and phase response of SMR.

The reflection coefficient *S*(1,1), impedance and phase response of SMR were measured with probe station and network analyzer and displayed in Fig. 5, respectively. A distinct resonant phenomenon was observed clearly, indicated the good quality of the SMR we fabricated. The frequency response of SMR with Ti/W Bragg reflector exhibited a return loss of −20 dB at the center of 2.22 GHz. There were no other resonances owing to shear modes and higher-order harmonics from the picture, which verifies that the Bragg reflector had a good effect on successfully restraining other frequency resonant and stopping the acoustic dissipation to the substrate.^35^ The series and parallel resonant frequencies of SMR appeared at 2.20 GHz and 2.23 GHz, respectively. Both the *k*_eff_^2^ and the *Q* of SMR can be derived easily by the expression from the *S*-parameter measurements as follows:^36^1
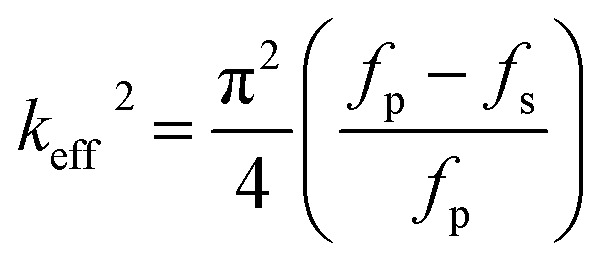
2
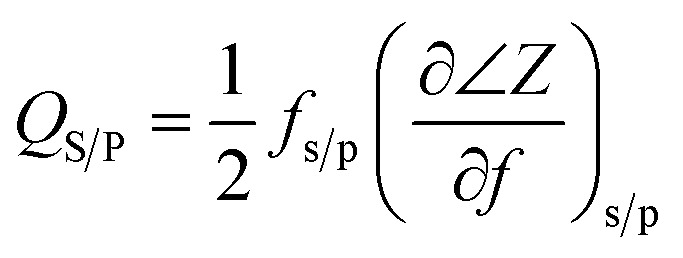
where *f*_p_ and *f*_s_ are parallel and series frequencies of SMR, *Q*_S_ and *Q*_P_ are parallel and series quality factors and *Z* is the input electrical impedance. According to calculation above, the *k*_eff_^2^, *Q*_S_ and *Q*_P_ of our SMR are 3.42%, 385, and 505, respectively.

**Fig. 6:**
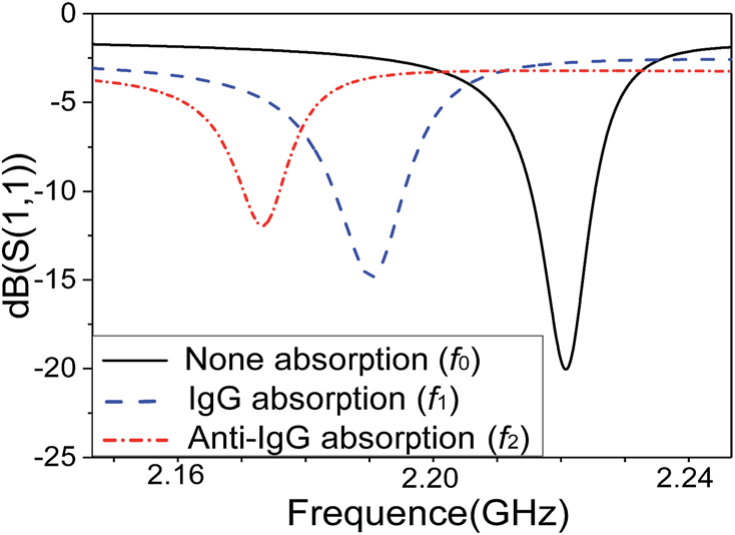
The resonant frequency shifts of the SMR after biomolecules adsorption.

Fig. 6 shows the resonant frequency shifts of the SMR after biomolecules adsorption, due to the mass loading effect. Prior to the mass-loading, the resonance peak *f*_0_ appeared at 2.220 GHz. The resonance peak *f*_1_ drifted down 2.190 GHz after absorbed human IgG antibody and BAS on the surface by the SAM method. Finally, the resonance peak *f*_2_ drifted down 2.170 GHz after binding goat anti-human IgG antigen with FITC on the surface. The result demonstrates clearly that the resonance frequency drifted down significantly by absorbing a small amount of biomolecules onto the surface due to the mass-loading effect confirming that the SMR can be used effectively as a sensor for biomolecule detections.

The sensitivity of SMR can be estimated conveniently by Sauerbrey's formula as follow:^37^3
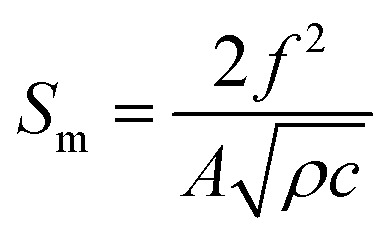
where *S*_m_ is the mass sensitivity, *f* is the unloaded resonance frequency, *ρ* is the AlN mass density, *A* is the area of operation and *c* is a stiffness constant of the AlN piezoelectric material. According to eqn (3), the goat anti-human IgG antigen sensitivity of our SMR sensor is estimated to be 3.15 kHz cm^2^ ng^−1^. The result demonstrates that our SMR sensor is highly promising in biomolecule detections because of its high sensitivity, small size, and low-cost than conventional QCM.^38,39^

**Fig. 7:**
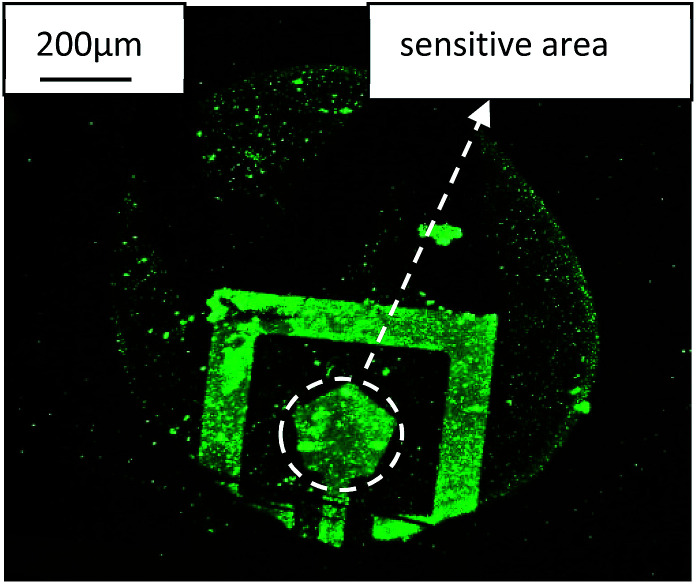
The FIM micrograph of the goat anti-human IgG antigen with FITC immobilized on the SMR surface.

In order verify the goat anti-human IgG antigen with FITC really immobilized on the Au electrode surface and caused a resonance frequency shift of SMR, the SMR sensor was observed by FIM and the micrograph was shown in Fig. 7. Nothing can be observed on the surface before the goat anti-human IgG antigen with FITC immobilized. After antigen immobilized, it is clearly observed that the top electrode was covered with fluorescent material that mainly consists of goat anti-human IgG antigen with FITC from the picture.

**Fig. 8:**
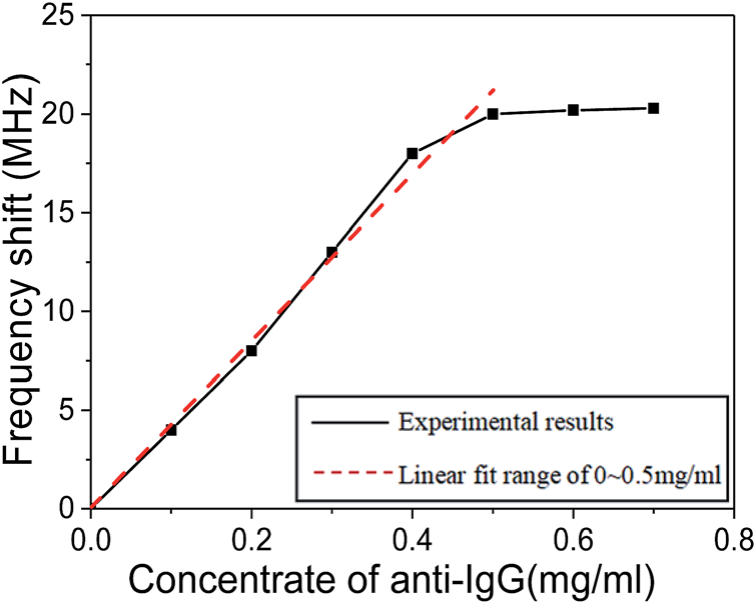
The relationship between the resonant frequency shift of SMR sensor and the concentration of goat anti-human IgG antigen.

The relationship between the resonant frequency shift of human IgG antibody immobilized SMR sensor and the concentration of goat anti-human IgG antigen ranging from 0 to 0.7 mg ml^−1^ is shown in Fig. 8. There was a nearly linear relationship between the resonant frequency shift and the concentration of goat anti-human IgG antigen from 0 to 0.4 mg ml^−1^. However, the frequency shift was relatively constant when the concentration of antigen was greater than 0.5 mg ml^−1^, indicating the attainment of antibody adsorption plateau. When the concentration of antigen was too high, there was not enough human IgG antibody on the electrode surface for binding, which is the reason why the resonant frequency shift was not obvious. As a result, 0.5 mg ml^−1^ of goat anti-human IgG antigen concentration was recommended as optimal concentration for SMR sensor.

**Fig. 9:**
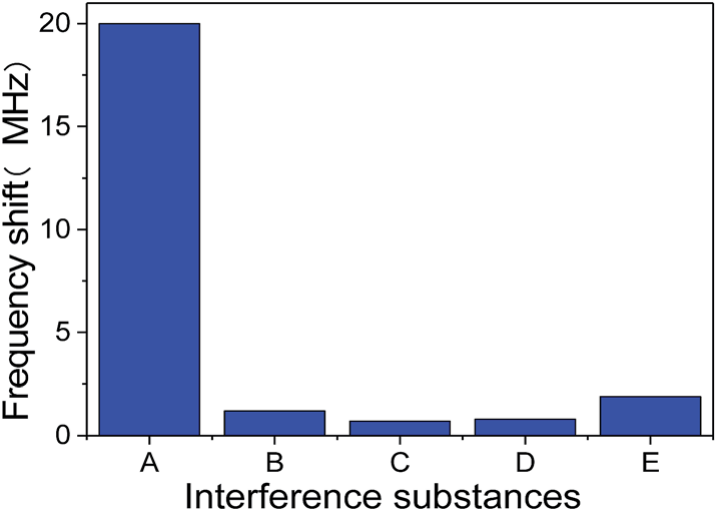
The response frequency shift of SMR sensor to other endogenous interference substances at 0.5 mg ml^−1^ concentration. (A is goat anti-human IgG, B is human IgG, C is goat anti-mouse IgG, D is goat anti-rabbit IgG and E is BSA).

In order to evaluate the specificity of SMR sensor, a series of interference experiments were also carried out. The human IgG antibody immobilized SMR was used to detect several endogenous substances such as human IgG, goat anti-mouse IgG, goat anti-rabbit IgG and BSA. The response frequency shift of SMR sensor to other endogenous substances were shown intuitively in Fig. 9. Adsorption goat anti-human IgG on human IgG antibody immobilized SMR surface resulted in a frequency shift down of 20 MHz, which demonstrates a higher sensitivity to target antigen. The introduction of human IgG, goat anti-mouse IgG, goat anti-rabbit IgG and BSA on human IgG antibody immobilized SMR surface only resulted in the frequency shift lower than 2 MHz, which indicates the non-specific bindings were negligible. These results suggest that the SMR sensor was able to discriminate goat anti-human IgG from other endogenous substances easily and the selectivity of SMR sensor was satisfactory.

## 4. Conclusions

In this study, we have fabricated a SMR sensor for detection of goat anti-human IgG antigen based on the specific binding between goat anti-human IgG antigen and human IgG antibody. The SMR consisting of a thin AlN piezoelectric film and Bragg acoustic reflector with a resonant frequency of 2.22 GHz, and the *k*_eff_^2^, *Q*_S_ and *Q*_P_ of SMR are 3.42%, 385 and 505, respectively. Human IgG antibody was immobilized on the electrode surface of SMR through SAM method for future detection. Then, goat anti-human IgG antigen with FITC was immobilized on the electrode surface of SMR through the specificity of bind between antibody and antigen and can be observed by FIM, clearly. The sensitivity of the SMR sensor for goat anti-human IgG antigen was estimated to be 3.15 kHz cm^2^ ng^−1^. The relationship between the resonant frequency shifts of SMR sensor and the concentrations of goat anti-human IgG antigen ranging from 0 to 0.7 mg ml^−1^ was investigated and 0.5 mg ml^−1^ was recommended as optimal concentration. A series of interference experiments were also applied to evaluate the specificity of SMR sensor and the result suggested the selectivity of sensor was satisfactory. Lastly, our SMR sensor provides a new approach for detection of goat anti-human IgG antigen. It also shows a promising future for biomolecule detections in medical diagnostics and environmental protections.

## Conflicts of interest

There are no conflicts to declare.

## Supplementary Material

## References

[cit1] Duan X., Li Y., Rajan N. K., Routenberg D. A., Modis Y., Reed M. A. (2012). Nat. Nanotechnol..

[cit2] Mu L., Droujinine I. A., Rajan N. K., Sawtelleet S. D., Reed M. A. (2014). Nano Lett..

[cit3] Kosaka P. M., Pini V., Ruz J. J., da Silva R. A., Ujue-González M., Ramos D., Calleja M., Tamayo J. (2014). Nat. Nanotechnol..

[cit4] Scarano S., Scuffi C., Mascini M., Minunni M. (2010). Biosens. Bioelectron..

[cit5] Guan W., Rajan N. K., Duan X., Reed M. A. (2013). Lab Chip.

[cit6] Ramachandran A., Wang S., Clarke J., Ja S. J., Goad D., Wald L. (2008). et al.. Biosens. Bioelectron..

[cit7] Altintas Z., Uludag Y., Gurbuz Y., Tothill I. (2012). Anal. Chim. Acta.

[cit8] Muren N. B., Barton J. K. (2013). J. Am. Chem. Soc..

[cit9] Voiculescu I., Nordin A. N. (2012). Biosens. Bioelectron..

[cit10] Yang Q., Pan S., Zhao Y., Zhang H., Pang W., Duan X. (2017). Biosens. Bioelectron..

[cit11] Zhao X., Pan F., Ashley G. M., Garcia-Gancedo L., Luo J., Flewitt A. J. (2014). et al.. Sens. Actuators, B.

[cit12] Wang T., Mu X., Randles A. B., Gu Y. D. (2015). Appl. Phys. Lett..

[cit13] RodríguezMadrid J. G., Iriarte G. F., Williams O. A., Calle F. (2013). Sens. Actuators, A.

[cit14] Erbahar D. D., Gurol I., Gümüş G., Musluoğlu E., Öztürk Z. Z., Ahsen V., Harbeck M. (2012). Sens. Actuators, B.

[cit15] Cheng C., Chang Y., Chu Y. (2012). Chem. Soc. Rev..

[cit16] Gill G. S., Prasad M. (2016). Sens. Lett..

[cit17] Johnston M. L., Kymissis I., Shepard K. L. (2010). IEEE Sens. J..

[cit18] Torres N. M. (2010). Sensors.

[cit19] Guo H., Gao Y., Liu T. (2018). Measurement.

[cit20] Fan L., Zhang S., Ge H., Zhang H. (2013). J. Appl. Phys..

[cit21] Piazza G., Stephanou P. J., Pisano A. P. (2006). J. Microelectromech. Syst..

[cit22] Guo P., Xiong J., Zheng D., Zhang W., Liu L., Wang S., Gu H. (2015). RSC Adv..

[cit23] He X., Garcia-Gancedo L., Jin P., Zhou J., Wang W., Dong S. (2012). et al.. J. Micromech. Microeng..

[cit24] Clement L. M., Delicado A., Olivares R. J., Mirea T., Sangrador J., Iborra E. (2017). Sens. Actuators, A.

[cit25] Wei C., Chen Y., Li S., Cheng C., Kao K., Chung C. (2010). Appl. Phys. A.

[cit26] Knapp M., Hoffmann R., Lebedev V., Cimalla V., Ambacher O. (2018). Nanotechnology.

[cit27] Satoh Y., Nishihara T., Yokoyama T., Ueda M., Miyashita T. (2005). Jpn. J. Appl. Phys..

[cit28] Rughoobur G., Sugime H., Demiguel-Ramos M., Mirea T., Zheng S., Robertson J. (2018). et al.. Sens. Actuators, B.

[cit29] Pang W., Zhao H., Kim E. S., Zhang H., Yu H., Hu X. (2012). Lab Chip.

[cit30] Han C., Chen D., Zhang Y., Xu D., Liu Y., Kong E. (2012). et al.. Nano-Micro Lett..

[cit31] Carrara S., Benini L., Bhalla V., Stagni C., Ferretti A., Cavallini A. (2009). et al.. Biosens. Bioelectron..

[cit32] Tsai W., Lin I. (2005). Sens. Actuators, B.

[cit33] Tang A., Pravda M., Guilbault G., Piletsky S., Turner A. (2002). Anal. Chim. Acta.

[cit34] Lin S., Huang J., Lii D. (2005). Mater. Chem. Phys..

[cit35] DeMiguel-Ramos M., Díaz-Durán B., Escolano J., Barba M., Mirea T., Olivares J. (2017). et al.. Sens. Actuators, B.

[cit36] Su Q., Kirby R., Komuro E., Imura M., Zhang Q., Whatmore R. (2001). IEEE Trans. Microwave Theory Tech..

[cit37] Sauerbrey G. (1959). Z. Phys..

[cit38] Wingqvist G., Anderson H. (2009). Biosens. Bioelectron..

[cit39] Fu Y. Q., Luo J. K., Du X. Y., Flewitt A. J., Li Y., Markx G. H. (2010). et al.. Sens. Actuators, B.

